# Evaluating Douglas Fir’s Provenances in Romania Through Multi-Trait Selection

**DOI:** 10.3390/plants14091347

**Published:** 2025-04-29

**Authors:** Emanuel Stoica, Alin Madalin Alexandru, Georgeta Mihai, Virgil Scarlatescu, Alexandru Lucian Curtu

**Affiliations:** 1 Department of Forest Genetics and Tree Breeding, “Marin Dracea” National Institute for Research and Development in Forestry, 077190 Voluntari, Romania; emanuel.stoica@icas.ro; 2 Department of Silviculture, Transilvania University of Brasov, 500123 Brasov, Romania; lucian.curtu@unitbv.ro; 3 National Institute for Research and Development in Forestry “Marin Dracea”, Mihăești Research Station, 117470 Mihaesti, Romania; virgils_ro@yahoo.com

**Keywords:** Douglas fir, common garden experiments, multi-trait selection, genotype–environment interaction, non-native tree species

## Abstract

Douglas fir (*Pseudotsuga menziesii* [Mirb.] Franco) is a valuable timber species native to western North America that was introduced to Europe in the 19th century. The objective of this study was to select the most valuable and stable Douglas fir provenances in Romania by combining growth and quality traits, using two indices recently used in forest tree species: the multi-trait genotype–ideotype distance index (MGIDI) and the multi-trait stability index (MTSI). The study was conducted across three common garden experiments in Romania, established in 1977, evaluating 61 provenances from the United States, Canada, Germany, France, and Romania. The analyzed traits were diameter at breast height (DBH), total height (TH), and pruned height (PH). Significant genotype–environment interactions were observed, with the Douglas fir showing superior growth performance in one of the testing sites in western Romania (Aleșd). The MGIDI and MTSI identified high-performing provenances from diverse geographic origins, including the Pacific Northwest, Europe, and Canada. Selection differentials ranged from 2.8% to 10.9% for individual traits, highlighting the potential for genetic improvement. The selected provenances represent valuable genetic resources of Douglas fir that are adapted to environmental conditions in the Carpathian region, contributing to the development of climate-adaptive breeding strategies and sustainable forest management.

## 1. Introduction

Douglas fir (*Pseudotsuga menziesii* [Mirb.] Franco) represents one of the most valuable timber species native to western North America. It is highly important for its remarkable wood and timber quality, rapid growth rate, and resistance to diseases and insects [1]. Douglas fir is ranked as the second-tallest tree species globally after *Sequoia sempervirens*; it can grow over 100 m in height, with trunks up to 4 m in diameter, and it lives for more than 1300 years in its native range [2].

Introduced from North America over 150 years ago, the Douglas fir has become one of the most economically significant non-native tree species in European forests [3]. Douglas fir was introduced to Europe between 1826 and 1827 by the Scottish botanist David Douglas and was subsequently spread in many European countries, with 80% of the total area concentrated in France, Germany, and the United Kingdom [1,2]. Douglas fir covers more than 823,000 ha across forests in 35 European countries, accounting for approximately 0.40% of the total forest area in Europe [4].

In Romania, the Douglas fir was first introduced in 1887 in the Moldavia region (Fântânele Forest District) situated in the northeast of the country. A year later, in 1888, a plantation of Douglas fir was established in Banat (Lugoj Forest District), located in the western part of Romania [5]. In Romania, it covers around 7300 ha, representing 0.12% of the forest area [4] and is considered to be the most valuable conifer species introduced in Romania [6,7].

In North America, two distinct geographic varieties of Douglas fir have been identified: the coastal variety, or green Douglas fir (*Pseudotsuga menziesii* var. *menziesii* [Mirbel] Franco) and the interior variety (*Pseudotsuga menziesii* var. *glauca* [Beissn.] Franco), also called the Rocky Mountain or blue Douglas fir [3]. The coastal variety has demonstrated superior adaptation to European conditions, exhibiting faster growth, greater frost resistance, and a higher resistance to fungal diseases, compared to the interior variety [1,3,8]. The native range of Douglas fir spans uneven distances, with the coastal variety extending over 2200 km from Central British Columbia along the Pacific Coast Ranges to central California, where it is primarily found in the western Sierra Nevada and northwestern California. The interior variety covers over 4500 km, stretching from central British Columbia along the Rocky Mountains to central Mexico [3,9]. Coastal Douglas fir grows at elevations from sea level to 1500 m, while the interior variety thrives at 500–3200 m, with temperature and moisture as the main limiting factors for their distribution [8].

Provenance trials are experiments in which trees from different geographic origins are grown in common environments to assess their adaptability under varying conditions [10]. The first common garden experiments for Douglas fir in Europe were established between 1910 and 1912 to compare seed sources from North America [1]. Regional tests were conducted initially, but a globally coordinated effort was launched by the International Union of Forest Research Organizations (IUFRO) to standardize trials and evaluate adaptation across different regions [11,12]. Research on Douglas fir genetic variability in Romania began in 1972 as part of the IUFRO provenance tests [6,13]. At 18 years, Douglas fir provenance tests in Romania showed high survival rates for natural seed sources from Idaho and central Washington (west of the Cascades) and excellent survival for Romanian provenances. Low-elevation sources from Washington and Oregon demonstrated the best height and volume growth, while local Romanian sources performed well but had more branches per whorl [1,14].

Forest tree breeding programs commonly use data from common garden experiments to guide the selection and breeding of tree populations, ensuring they are adapted to their planting environments and improving productivity and resilience [15,16]. Genotype and environment (GxE) interactions in common garden experiments are critical for understanding how different tree species respond to varying environmental conditions [16]. These interactions can significantly influence quantitative and quality traits. Studies indicate that tree provenances show varying responses to environmental conditions, which suggests that selection at the provenance level is crucial for optimizing growth traits [17,18]. Significant GxE interactions were noted across different European locations for Douglas fir [19,20]. In particular, studies have shown that coastal provenances of Douglas fir tend to outperform interior ones under a variety of environmental conditions, suggesting that provenance selections are essential [20]. Overall, understanding these interactions is necessary for effective breeding programs, forest management, and conservation strategies, especially as climate change continues to impact forest ecosystems [21,22]. In the context of climate change, the successful adaptation of Douglas fir from its native maritime climate in the Pacific Northwest of North America to Romania’s continental climate suggests a promising capacity for climate resilience, particularly important for the next strategies in the breeding program. The adaptive capacity of tree species can be assessed through phenotypic plasticity, which refers to a genotype’s capacity to express various phenotypes under different environmental conditions.

Multi-trait selection in forest tree species has evolved significantly over the decades. Multi-trait selection can be used in a breeding program and simultaneously considers multiple traits to identify superior genotypes. For example, selection may include both growth and quality traits [23]. This approach enables breeders to make balanced decisions by evaluating important characteristics at once, rather than focusing on a single trait. Multi-trait genomic selection has been applied to improve biomass yield, growth rate, and wood quality in *Populus* breeding programs [24], but it has also been successfully implemented in *Eucalyptus robusta* breeding programs, improving the accuracy of genotype predictions and achieving significant genetic gains for growth and wood quality traits [25]. Some studies have expanded multi-trait selection approaches to include species such as birch [26], beech [27], and larch [28].

Recent studies include the application of multi-trait evaluations of selection and stability analysis in *Picea abies* [29] and multi-trait evaluations of height, basal area, and stem quality in *Fagus sylvatica* [23], *Populus* species [30], and *Pinus elliotti* [31]. These studies point out the progression of methodologies and the growing emphasis on multi-trait selection to enhance forest tree species adaptability and productivity.

The aim of this study was to select the most valuable and stable Douglas fir provenances by combining growth (total height and diameter at breast height) with quality traits (pruned height). This selection process was based on two indices: the MGIDI—multi-trait genotype–ideotype distance index [32] and the MTSI—multi-trait stability index [33]. The specific objectives of the study were as follows: (1) to select Douglas fir provenances with high performance using multiple traits in each trial site, (2) to examine genotype–environment interactions, (3) to investigate the stability of the provenances selected, and (4) to select the high-performance and stable Douglas fir provenances through multiple traits. The results will facilitate the improvement of Douglas fir by identifying provenances that demonstrate superior growth traits (above average values) with stable performance across different environmental conditions. Furthermore, the results will support more efficient reforestation efforts by providing a reliable basis for selecting provenances that offer both high productivity and environmental adaptability.

## 2. Materials and Methods

### 2.1. Experimental Design

**Figure 1:**
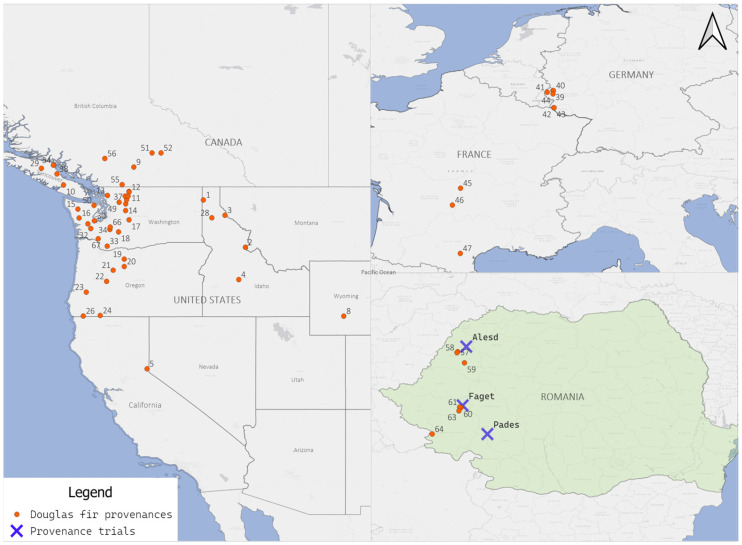
Geographic location of the tested Douglas fir provenances (red points) and common garden experiments (blue X).

In Romania, five common garden experiments with Douglas fir provenances were established between 1977 and 1980, of which three still exist. The three common garden experiments are located in Aleșd, Făget, and Padeș (Figure 1) and were established in 1977 [13]. These trials were installed in the distinct geographic regions of Romania: west (Banat), northwest (Crișana), and southwest (Getic Plateau). The selection of these areas was based on the consideration that they would provide favorable conditions for Douglas fir. The Douglas fir provenances tested in the trials were collected from a variety of geographic regions, including 34 provenances from the United States (Idaho—5, California—2, Oregon—6, Washington—20, Wyoming—1) and 10 from Canada, as well as 6 from Germany, 3 from France, and 8 from Romania (Figure 1). Details on the Douglas fir provenances are in the Supplementary Materials (Table S1).

In their natural range, the 61 provenances are distributed differently across the three common garden experiments. The sampling process included provenances located at diverse latitudes, ranging from 39°30′ N in California to 50°50′ N in Canada, and longitudinally, from 108°08′ W in Wyoming to 126°00′ W on the western coast of Vancouver Island, bordering the Pacific Ocean. The altitudinal range is from 60 m to 2438 m. The number of provenances tested in the common garden experiments has varied: 55 in Aleșd, 49 in Făget, and 48 in Padeș. In all three common garden experiments, 38 provenances are common (Table 1).

Mean climate values were calculated for the period 1997-2023 using ClimateEU [34]. Precipitation and the mean temperature are presented by year and for the growing season in parentheses (May to September).

The three common garden experiments, established in 1977 were planted in a partially balanced block design. Each provenance was represented by 25 individuals (5 × 5) within a plot, with 2 × 2 m spacing in three blocks.

The measurements were carried out in the summer of 2024, 47 years after planting. The traits chosen to assess the adaptive variation were diameter at breast height (DBH), total height (TH), and pruned height (PH). The pruned height was defined as the height of the lowest green branch. Trees with a higher pruned height tend to have fewer branches, resulting in wood with fewer knots, which is desirable for timber production. Therefore, the natural pruning ability of Douglas fir is associated with wood quality. Total and pruned height were measured using a Vertex IV with a precision of 0.1 m, and the diameter at breast height was measured using a caliper with a precision of 0.1 cm. These traits are critical variables in tree breeding and provenance studies, as they collectively provide insights into growth potential [35], wood quality [36], climate-change adaptation [37], and forest dynamics [38], enabling the selection of superior provenances and the development of sustainable forest management strategies.

### 2.2. Statistical Analysis

The quantitative genetic analysis of phenotypic variation was conducted both within each trial site and across sites. At each experimental site, a mixed linear model was used, wherein the provenance factor was considered a random effect, while the block effect was integrated into the model as a fixed effect [39]:

Yjkl = μ + Pj + Bk + Pj × Bk + ejkl(1)

where Yjk is the observed value of traits (DBH, HT, PH), μ is the general mean, Pj is the effect of the jth provenance, Bk is the effect of the kth block, Pj × Bk is the provenance-by-block interaction, and ejkl is the error term associated with ejkl trees (j is the provenance, k is the block, and l is the individual observation).

The statistical analyses were implemented through the lmerTest R package, which facilitates the computation and testing of linear mixed effects models [40].

The analysis conducted across sites was based on a mixed linear model, where both provenance and the interaction between provenance and site were examined as random effects. In contrast, the site was considered to be a fixed effect in the model:

Yijkl = μ + Si + Pj + Bk + Si × Pj + eijkl(2)

where Yijk is the observed value of traits (DBH, HT, PH), μ is the general mean, Si is the effect of the ith site, Pj is the effect of the jth provenance, Bk is the effect of kth block, Si × Pj is the provenance-by-site interaction, and eijkl is the error term associated with ijkl trees (i is the site, j is the provenance, k is the block, and l is the individual observation).

This approach allowed for the evaluation of genetic variance associated with provenance, as well as the potential variability introduced by different environmental factors across sites. By considering the provenance x site interaction as a random effect, the model accounted for potential site-specific variation in traits, providing a more comprehensive understanding of genetic and environmental influences.

To assess the stability and adaptability of Douglas fir provenances of different geographic origins, these two factors were evaluated across multiple traits using two recently introduced selection indices. The multi-trait genotype–ideotype distance index (MGIDI) [32] was applied to each trial site, while the multi-trait stability index (MTSI) [33] was used for evaluating performance and stability across sites. Both indices were implemented through the *metan* R package [41], providing a comprehensive tool for examining the interaction between genetic and environmental conditions. The selection intensity in each trial site for the MGIDI was 15%, with 8 provenances out of a total of 55 at Aleșd, 7 out of 48 at Padeș, and 7 out of 49 at Făget.

The MGIDI was calculated in four steps:

(1) Rescaling traits: values were scaled to 0–100, with higher scores indicating desired increases or decreases depending on the trait.
(2) Factor analysis: traits were grouped into factors based on correlations between them, and scores were assigned for each provenance.
(3) Defining the ideotype: an ideal genotype was set with a value of 100 for all traits
(4) Calculating distances: the distance between each genotype and the ideotype was computed to derive the MGIDI [32].

The MGIDI is calculated with the following formula:

(3) $${MGIDI}_{i}={\left[\sum_{j=1}^{f}{\left(y_{ij}-y_{j}\right)}^{2}\right]}^{0.5}$$

where *MGIDI_i_* is the multi-trait genotype–ideotype distance index for the *i*th genotype, *y_ij_* is the score of the *i*th genotype in the *j*th factor, and *y_j_* is the *j*th score of the ideotype.

The MTSI was calculated in four steps:

(1) Rescaling values: both the WAASB (Weighted Average of Absolute Scores, measuring genotype stability across environments using linear mixed-effect models) and trait performance values were scaled to a uniform range of 0 to 100 (0 represents the minimum and 100 represents the maximum).
(2) Calculating the WAASBY index: the WAASBY index was derived to balance stability (WAASB) and mean performance (Y), with the flexibility to adjust weights to emphasize either stability or performance [33].
(3) Defining the ideotype: similar to the MGIDI, but in this case, the ideotype was assigned a value of 100 for the WAASBY index to represent the ideal combination of stability and performance.
(4) MTSI estimation: the MTSI value was then calculated using the following formula:

(4) $${MTSI}_{i}={\left[{\sum_{j=1}^{f}(F_{ij}-F_{j})}^{2}\right]}^{0.5}$$

where the *MTSI_i_* = the multi-trait stability index for the *i*th genotype; F = the score; *F_ij_* = the *j*th score of the *i*th genotype; *Fj* = the *j*th score of the ideotype.

## 3. Results

### 3.1. Genetic Variability Analysis for Trial Sites

Analysis of random and fixed effects for Douglas fir across common garden experiments revealed distinct patterns of genetic and environmental variability (Table 2). The likelihood ratio test (LRT) results indicated significant provenance effects with varying intensity levels across trials and traits.

In Aleșd, provenance effects were significant for all analyzed traits (*p* ≤ 0.001), with LRT values ranging from 23.16 to 29.67. The provenance variance component (Vp) showed substantial values for DBH (7.23) and TH (3.36), accounting for 10.63% and 14.58% of the total variance, respectively. Block effects (MS b) were non-significant for DBH and TH but highly significant for PH.

Făget showed high heterogeneity in block effects with substantial mean square values (ranging from 565.59 to 923.91). Provenance effects remained significant for all traits. The proportion of variance explained by provenance ranged from 4.86% for DBH to 12.55% for PH.

In contrast, Padeș showed a distinct pattern, with non-significant provenance effects for DBH, but significant effects for TH and PH. Provenance variance components were lower (Vp between 0.38 and 1.93), representing between 3.65% and 9.42% of the total variance. Block effects were non-significant for all traits.

### 3.2. Genotype by Environment Analysis

Analysis of growth traits across three common garden experiments revealed significant genotype x environment interactions for all traits (Table 3). Among the common garden experiments, Aleșd recorded the highest average DBH per experiment ($\bar{x}$ = 35.73 cm) and total height ($\bar{x}$ = 29.94 cm).

Residual variance dominated across all traits, accounting for 90–94% of the total variance, while genotype x environment interaction variance (Vge) contributed 6–9%. Provenance effects (LRTg) were non-significant across all traits. Environmental effects (MS Env) were highly significant for all traits, with particularly strong effects on DBH. Block effects within environments were highly significant for both DBH and TH.

### 3.3. Multi-Trait Selection in Each Provenance Trial

Based on the MGIDI selection, performant provenances were identified across all three trial sites, with 8 provenances selected from 55 at Aleșd, 7 from 49 at Făget, and 7 from 48 at Padeș. This selection represented 15% of the best-performing provenances at each trial, maintaining a consistent selection intensity across all trials while accounting for the different total numbers of provenances tested at each location. The selected provenances consistently outperformed site means across all studied traits, with selection differentials ranging from 2.8% to 10.9%, highlighting the success of the selection process (Table 4).

The provenances selected at Aleșd covered three countries. From Romania, the selected provenances were as follows: 59—Toplița, 62—Vîrful Dăii, and 63—Nădrăgel. From the United States, the selected provenances were as follows: 2—Idaho, 20—Benton (Corvallis), 26—Siskiyou (Hawkinsville), and 31—Hoodsport, representing a range of environments from the Pacific Northwest to the mountain regions of Idaho. Finally, provenance 11—Duncan was from Canada.

The provenances selected at the Făget trial were predominantly from the United States with one notable European representative. From the United States, the selections included 2—Idaho, representing inland mountain populations, and several Pacific Northwest provenances: 5—El Dorado, 14—Snohomish (Sloan Creek), 21—Marion Forks, 22—Yacolt (Spotted Deernt—Battle G.), and 50—Clallam Country (Louella), plus 41—(Prüm Süd—Abt. 79 C—Reservation) from Germany.

The provenances selected at the Padeș trial included 59—Toplița from Romania. The selected provenances from the other European countries were: 46—Les Farges III from France, and 44—Manderscheid (Abt. 36 B2—Reservation) from Germany. The United States was represented by 31—Elma, (Washington), 34—Vicinity (Mineral Walker Road), 37—Concrete (Presentin Creek), and 23—Rosenburg, covering a range of ecological zones from coastal to inland regions of the Pacific Northwest.

### 3.4. The Multi-Trait Selection Across Common Garden Experiments

**Figure 2:**
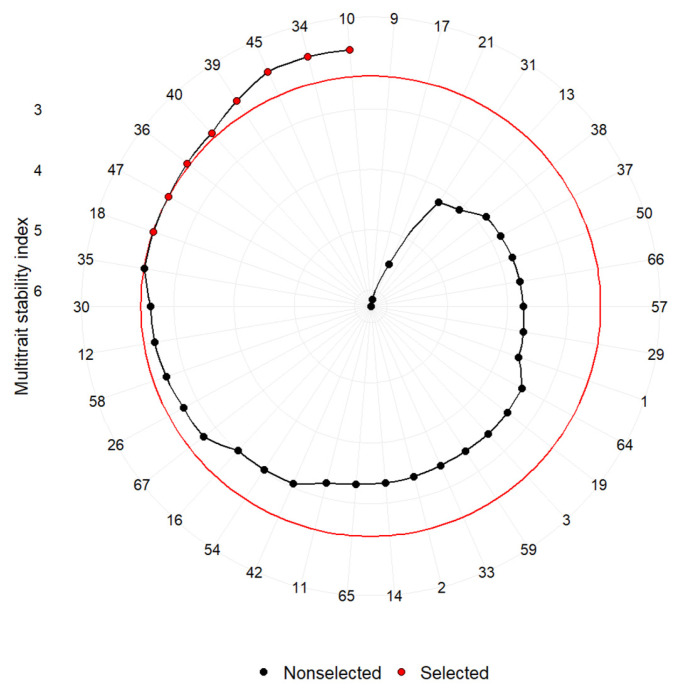
Ranking and selection of Douglas fir provenances based on MTSI with a 20% selection intensity (red line). The dots represent the multi-trait stability index. Lower values of the MTSI for a provenance indicate that it is closer to the ideotype.

Based on the performance and stability of multiple traits—DBH, total height, and pruned height—integrated within the MTSI, the following eight provenances were selected as the most suitable across the common gardens experiments: 36—Skycomish (Beckler Peak), 34—Vicinity (Mineral Walker Road), and 18—Lewis (Packwood) from the USA; 40—Daun Ost (Abt. 46 C), 39—Daun Ost (Abt. 39 A), 45—Poinsat (Puy de Dome) and 47—Moussans II from France; and 10—Franklin River (British Columbia) from Canada (Figure 2).

**Figure 3:**
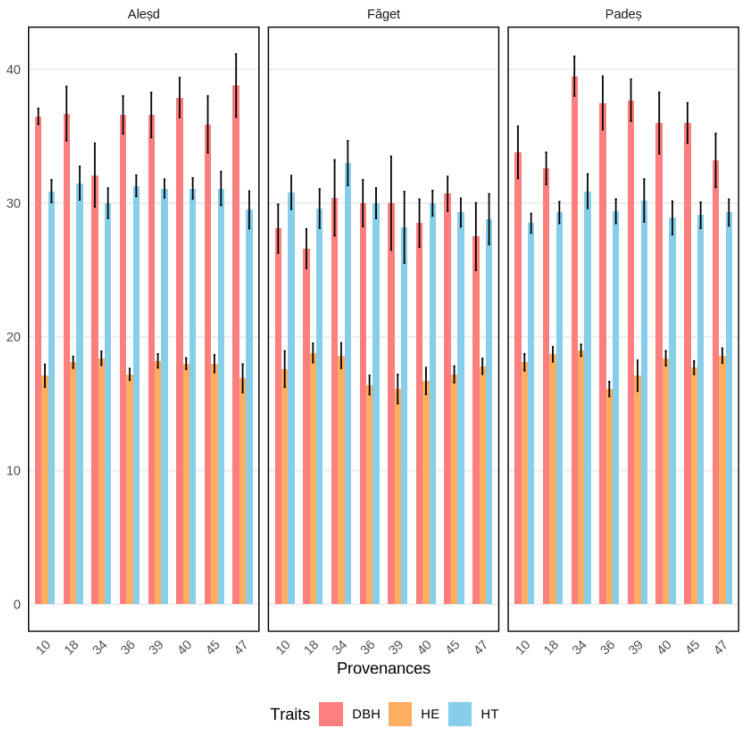
Mean trait value across common garden experiments; DBH—diameter at breast height, HE—pruned height, and HT—total height for the best performing provenances. Whiskers represent the standard error.

They also represent diverse ecological origins: the Pacific Northwest (36—Skycomish, 34—Vicinity, 18—Lewis, Franklin River), Central Europe (39—Daun Ost, Abt. 39 A and 40—Daun Ost, Abt. 46 C), French mountainous regions (45—Poinsat, 47—Moussans II), and Canada (10—Franklin River) (Figure 3).

The analysis of selection differentials using the MTSI for DBH reveals a notable pattern across common garden trials. The Aleșd trial showed the highest genetic gain potential of 10%, while Făget and Padeș showed more moderate improvements of 5.2% and 3.2%, respectively (Table 5).

Regarding total height, the selection response was more uniform across trials, with Aleșd achieving a 5.6% gain, followed closely by Făget at 4.4% and Padeș at 4.2%.

For pruned height, Făget emerged as the standout location with a selection differential of 6.9%, significantly outperforming Aleșd and Padeș, which showed modest gains of 2.9% and 2.8%, respectively.

## 4. Discussion

The results of this study provide valuable insights into the genetic variability and adaptation potential of Douglas fir in Romania, with important implications for breeding programs and forest management. The analysis of genetic variability within trial sites revealed significant provenance effects with varying intensities across sites and traits. The selected provenances demonstrated superior performance compared to site means across all evaluated traits, highlighting the effectiveness of the selection process (Figure 3).

The analysis of random and fixed effects across the three common garden experiments revealed distinct patterns of genetic control and environmental influence. For the Aleșd trial, strong provenance effects were significant for all analyzed traits, particularly for DBH and TH, which aligns with recent findings of site-specific genetic expression in Douglas fir adaptation [42]. The Făget trial demonstrated high heterogeneity in block effects, with substantial mean square values ranging from 565.59 to 923.91, suggesting the effect of microsite conditions on growth variability [43]. In contrast, the Padeș trial showed non-significant provenance effects for DBH, but maintained significance for height traits, suggesting complex interactions between genetic and environmental factors. The selection response for total height demonstrated remarkable consistency across trials. This uniformity in height growth suggests a stable pattern of adaptation among the Douglas fir provenances, regardless of specific environmental conditions.

The significant genotype x environment interactions observed for all traits emphasized the need for selecting provenances that are specifically adapted to different environmental conditions. These interactions showed that growth performance varied substantially across different environmental conditions, suggesting that genotypes responded differently to specific site conditions. Similar findings were reported in a study on coastal Douglas fir provenances, which demonstrated the critical role of genetic correlation in forest tree breeding [44]. The dominance of residual variance (90–94%) in the GxE analysis further underlines the influence of uncontrolled environmental factors on growth, a challenge also noted in recent North American studies on assisted migrations [45].

The variation in growth performance across common garden experiments suggests that Douglas fir’s adaptation to Romanian conditions involves both genetic factors and phenotypic plasticity, as evidenced by the different height gain percentage observed at each location. Phenotypic plasticity is a fundamental mechanism that allows forest tree species to adapt to different environmental conditions [46].

Multi-trait selection using the MGIDI and MTSI successfully identified superior provenances from diverse origins, including the Pacific Northwest, Europe, and Canada. These results are consistent with findings from rotation-age growth performance studies, which highlight the genetic diversity and adaptability of Douglas fir provenances [20].

The selection differentials in our study (2.8–10.9%) are consistent with the recent forest tree breeding literature: Alexandru et al. [29] reported 0.2–17.8% for Norway Spruce, while Liepe et al. [23] found 5.2–17.9% gains for height and basal area in the European beech.

The estimated genetic gains, particularly for DBH in the Aleșd trial (10%), are promising for future breeding programs. These findings emphasize the dual benefits of genetic improvement for both growth performance and economic returns [47]. Selection differentials, which measure the effectiveness of selecting superior provenances, have been reported to range from 0.2% to 17.8% greater than site means, indicating that certain provenances consistently outperform others in specific environments. The Romanian Douglas fir provenances of 59—Toplița, 62—Vârful Dăii, and 63—Nădrăgel were selected based on their performance in specific trial sites using the MGIDI. Provenances 62—Vârful Dăii and 63—Nădrăgel were selected at the Aleșd trial site, where the average DBH was 35.73 cm and the TH was 29.94 m. These provenances demonstrated superior growth traits, with selection differentials ranging from 2.8% to 10.9%, highlighting their adaptability to local conditions. Provenance 59—Toplița, selected at the Padeș trial site, showed a stable performance, particularly in terms of total height and pruned height, despite non-significant effects for DBH.

Douglas fir demonstrated superior growth characteristics at Aleșd, with mean DBH values 15% being higher than at Făget, and 23% higher than at Padeș, suggesting optimal site conditions for Douglas fir development. Our results suggested that site conditions significantly influence the expression of genetic potential in Douglas fir, with implications for provenance selection and strategies. The strong performance at Aleșd, characterized by both superior growth and higher genetic variance components, suggests that matching provenances to optimal sites maximize genetic gain potential [48].

There are reports showing that pruning Douglas fir can significantly influence wood quality and growth characteristics [49]. Our results show significant provenance effects for pruned height (PH) across Aleșd (14.58%), Făget (12.55%), and Padeș (9.42%). Reports indicate that pruning the lower one-third of the live crown does not adversely affect growth, supporting the adaptability of provenances with higher PH values to maintain productivity [50]. Additionally, the positive correlation between juvenile tree heights and breast height diameters over time reinforces the importance of PH as a predictor of long-term growth and wood quality [51]. These findings suggest that PH not only reflects genetic and environmental adaptability but also serves as an essential measure for optimizing pruning strategies to enhance timber quality without compromising growth.

In the context of climate change, the genetic diversity identified in this study represents a valuable resource for adapting Douglas fir to changing environmental conditions. The selected provenances, with their diverse ecological origins and demonstrated adaptation potential, can significantly contribute to the development of more resilient and locally adapted tree populations. These findings are particularly relevant for developing climate-adaptive breeding strategies and optimizing forest management practices.

This study is limited by the assessment of only three traits, which reduces the ability to evaluate other important adaptive and quality characteristics of Douglas fir. Future research should focus on the long-term monitoring of selected provenances, with a particular focus on drought tolerance, wood quality, and pest resistance. More studies would help to validate and extend our findings, thereby reinforcing the role of Douglas fir as a source of forest reproductive material in the context of climate change.

## 5. Conclusions

Common garden experiments have a key role in evaluating the genetic variability and adaptability of Douglas fir provenances. They not only reveal which genetic resources are best suited to local conditions in Romania, but also provide a comprehensive framework for sustainable forestry by identifying provenances with superior growth and quality traits.

The study demonstrates that the use of multi-trait selection methods is effective in integrating complex growth parameters. By simultaneously considering traits such as basal diameter, total height, and pruned height, the approach makes a more robust selection process possible, ensuring that the chosen provenances possess a balanced performance that meets both economic and ecological criteria.

Substantial genotype–environment interactions were observed, indicating that individual provenances respond differently depending on local site conditions. This variation emphasizes the need for localized selection strategies, which can help to optimize reforestation efforts and improve overall forest resilience.

The implementation of new indices like the MGIDI and MTSI has proven instrumental in quantifying selection differentials and identifying provenances with significant genetic improvement potential. These metrics not only provide precise comparisons across diverse provenances but also highlight the potential for implementing advanced selection techniques.

Overall, the Douglas fir provenances tested in Romania demonstrate a high potential for climate adaptation and contribute to the development of targeted breeding programs, paving the way for sustainable forest management that effectively addresses the challenges posed by current environmental changes.

## Figures and Tables

**Table 1:** Characteristics of geographic location, climate, and soil of the three trial sites.

	Aleșd	Făget	Padeș
Coordinates	47.15° N, 22.33° E	45.76° N, 22.29° E	45.09° N, 22.89° E
Temperature (°C) (1997–2023)	7.9 (15.3)	10.4 (17.9)	7.82 (15.4)
Precipitation (mm) (1997–2023)	71.6 (90.0)	70.9 (90.9)	81.25 (100.5)
Elevation (m a.s.l.)	670	330	720
Soil	Eutricambosoil	Luvosol	Eutricambosoil

**Table 2:** Results of random and fixed effects for three traits in each Douglas fir provenance trial.

Trial	Trait	LRTp	Vp	Vr	MS b	Mean ± SD
Aleșd	DBH (cm)	23.16 ***	7.23	60.77	80.66 ns	36.05 ± 8.18
	TH (m)	29.67 ***	3.36	19.68	13.32 ns	30.36 ± 4.71
	PH (m)	24.55 ***	0.98	7.34	228.08 ***	17.48 ± 2.91
Făget	DBH (cm)	8.27 **	2.87	56.16	565.59 ***	28.82 ± 7.75
	TH (m)	12.80 ***	2.009	24.73	923.91 ***	29.61 ± 5.31
	PH (m)	34.01 ***	1.73	12.05	735.91 ***	17.32 ± 3.86
Padeș	DBH (cm)	2.89 ns	1.93	50.89	0.31 ns	34.71 ± 7.25
	TH (m)	18.97 ***	1.59	15.29	20.03 ns	28.77 ± 4.1
	PH (m)	7.78 **	0.38	6.74	20.02 ns	17.68 ± 2.67

** indicates significance at *p* ≤ 0.01, and *** at *p* ≤ 0.001; ns: not statistically significant with *p* > 0.05; LRTp—likelihood ratio test for provenance effect; Vp—variance for provenance random effect; Vr—residual variance; MS—mean square values for block (b); DBH—diameter at breast height; TH—total height; PH—pruned height.

**Table 3:** Results of random and fixed effects for four traits of the Douglas fir provenances.

Traits	LRTg	LRTge	Vp	Vge	Vr	MS Env	MS B (Env)	Mean ± SD
DBH (cm)	0 ns	20.74 ***	3.53 (27%)	3.91 (30%)	5.69 (43%)	1196 ***	70.96 ns	33.03 ± 8.43
TH (m)	0 ns	28.03 ***	0 (0%)	1.75 (8%)	20.36 (92%)	221.8 ***	162.9 ***	29.50 ± 4.79
PH (m)	0.03 ns	38.20 ***	0.03 (1%)	0.92 (9%)	8.97 (90%)	57.88 **	161.7 ***	17.47 ± 3.24

** indicates significance at *p* ≤ 0.01, and *** at *p* ≤ 0.001; ns: not statistically significant with *p* > 0.05; LRTg—likelihood ratio test for provenance effects; LRTge—likelihood ratio test for genotype x environment interaction; Vp—variance for provenance random effect; Vr—residual variance; MS—mean square values for block (b); DBH—diameter at breast height; TH—total height; PH—pruned height; values in brackets report the proportion of variance accounted for by each effect.

**Table 4:** Selection differentials by trial site using the MGDI for Douglas fir provenances.

Traits	Site Mean			Selected Provenances Mean			Selection Differential %		
	Aleșd	Făget	Padeș	Aleșd	Făget	Padeș	Aleșd	Făget	Padeș
DBH (cm)	35.9	28.8	34.7	39.8	30.3	35.8	10.9	5.2	3.2
TH (m)	30.2	29.6	28.8	31.9	30.9	30	5.6	4.4	4.2
PH (m)	17.3	17.3	17.7	17.8	18.5	18.2	2.9	6.9	2.8

DBH—diameter at breast height; TH—total height; PH—pruned height.

**Table 5:** Selection differentials by trial site using the MTSI for Douglas fir provenances.

Traits	Site Mean			Selected Provenances Mean			Selection Differential %		
	Aleșd	Făget	Padeș	Aleșd	Făget	Padeș	Aleșd	Făget	Padeș
DBH (cm)	35.9	28.8	34.7	37.1	29.62	36.75	3.3	2.8	5.9
TH (m)	30.2	29.6	28.8	30.92	30.22	29.5	2.4	2.1	2.4
PH (m)	17.3	17.3	17.7	17.82	17.62	17.8	3.0	1.8	0.6

DBH—diameter at breast height; TH—total height; PH—pruned height.

## Data Availability

The data presented in this study can be obtained from the corresponding authors upon request.

## Supplementary Materials

The following supporting information can be downloaded at: https://www.mdpi.com/article/10.3390/plants14091347/s1.
