# Supplementary material for: Evaluating Douglas Fir’s Provenances in Romania Through Multi-Trait Selection
Source: Plants (Basel). 2025 Apr 29;14(9):1347. doi: 10.3390/plants14091347 (PMC12073550; doi:10.3390/plants14091347)
Supplement: Supplementary file 1 [file plants-14-01347-s001.zip › plants-3553716-supplementary.pdf]

Table S1. Details about the tested Douglas fir provenances

No. of provenance	Name of provenance	Country	Lat. N	Long. E	Altd. (m)
1	Idaho (var. *glauca*)	SUA	48°00′	-116°95′	780
2	Idaho (var. *glauca*)	SUA	47°48′	-113°61′	1389
3	Idaho (var. *glauca*)	SUA	47°52′	-115°68	1356
4	Boise (*var. galuca*)	SUA, Idaho	44°00′	-115°00′	1829
5	El Dorado	SUA California	39°17′	-120°08′	1387
8	South End Pass	SUA, Wyoming	50°07′	-120°85′	900
9	Merrit	Canada, Columbia Britanică	50°04′	-120°51′	900
10	Franklin River	Canada, Columbia Britanică	49°06′	-124°46′	150
11	Duncan	Canada, Columbia Britanică	48°75′	-121°17′	450
12	Diablo Dam – Whatcom	SUA, Washington	48°72′	-121°17′	450
13	Skagit – Sedro Wooley	SUA, Washington	48°53′	-122°31′	60
14	Snohomish -Sloan Creek	SUA, Washington	48°08′	-121°18′	650
15	Jefferson – Hoh River	SUA, Washington	47°48′	-123°58′	240
16	Grays Harbour – Humptulips	SUA, Washington	47°19′	-123°54′	140
17	Kittias – Cle Elum	SUA, Washington	47°13′	-121°07′	640
18	Lewis – Packwood	SUA, Washington	46°34′	-121°42′	300
19	Pine Grove	SUA, Oregon	45°06′	-121°23′	730
20	Benton – Corvallis,	SUA, Oregon	44°42′	-121°23′	76
21	Marion Forks	SUA, Oregon	44°30′	-122°00′	1060
22	Lane – Oakridge	SUA, Oregon	43°54′	-122°22′	880
23	Rosenburg	SUA, Oregon	43°19′	-123°30′	280
24	Jakson – Ashland	SUA, Oregon	42°05′	-122°39′	1500
26	Siskiyou – Hawkinsville,	SUA, California	41°47′	-123°40′	1060
28	Chatcolet, S., (var. *galuca*)	Idaho, SUA	47°20′	-116°30′	700
29	Vancouver	Canada, Columbia Britanică	50°00′	-126°00′	150
30	Hoodsport	SUA, Washington	47°10′	-123°03′	300
31	Elma, Washington	SUA, Washington	47°00′	-123°30′	300
32	Pe Ell-Sand Creek, Mc. Donald	SUA, Washington	46°45′	-123°15′	200
33	Yacolt – Spotted Deernt – Battle G.	SUA, Washington	45°48′	-122°20′	600
34	Vicinity, Mineral Walker Road	SUA, Washington	46°40′	-12°15′	500
35	Darrington – Texas Pond	SUA, Washington	48°18′	-121°15′	280
36	Skycomish – Beckler Peak	SUA, Washington	47°42′	-121°20′	500
37	Concrete – Presentin Creek	SUA, Washington	48°30′	-121°20′	110
38	Oyster R.	Canada, Columbia Britanică	49°42′	-125°08′	400
39	Daun Ost – Abt. 39 A – Rezervație	Germania	50°12′	-06°50′	520
40	Daun Ost – Abt. 46 C.	Germania	50°11′	-06°52′	500
41	Prüm Süd – Abt. 79 C – Rezervație	Germania	50°13′	-06°25′	380
42	Wittlich West – Abt. 3B	Germania	49°47′	-06°54′	230
43	Wittlich West – Abt. 1B	Germania	49°47′	-06°55′	230

44	Manderscheid – Abt. 36 B2 – Rezervație	Germania	50°06′	-06°50′	400
45	Poinsat – Puy de Dome	Franța	46°04′	-02°43′	750
46	Les Farges III	Franța	45°32′	-02°07′	665
47	Moussans II	Franța	43°26′	-02°42′	750
49	Darrington III	SUA, Washington	48°10′	-121°40′	200
50	Clallam Contry – Louella	SUA, Washington	48°00′	-123°04′	330
51	Pinetan	Canada, Columbia Britanică	50°50′	-119°50′	830
52	Shuswape Lake	Canada, Columbia Britanică	50°50′	-119°20′	530
54	Morton Lake	Canada, Columbia Britanică	50°10′	-125°20′	150
55	Centre Creek – Chilliwack Valey	Canada, Columbia Britanică	49°07′	-121°30′	460
56	Devine – Dist.	Canada, Columbia Britanică	50°32′	-122°28′	380
57	Pădurea Neagră	România	47°03′	22°17′	500
58	Piatra Albă	România	47°01′	22°15′	580
59	Toplița	România	46°46′	22°20′	330
60	Aninoasa Mare	România	45°43′	22°15′	550
61	Vîrful Dăii	România	45°44′	22°13′	825
62	Vîrful Dăii	România	45°44′	22°13′	420
63	Nădrăgel	România	45°43′	22°13′	560
64	Anina – Buhui	România	45°10′	21°55′	650
65	Sedro Wooley	SUA, Washington	48°30′	-122°10′	720
66	Elbe	SUA, Washington	46°50′	-122°10′	720
67	Kelso	SUA, Washington	46°12′	-122°50′	609
